# Results of orthodontic procedure in a patient with classic infantile Pompe disease

**DOI:** 10.1186/s13052-025-02023-6

**Published:** 2025-07-15

**Authors:** Carla Maria Grimaldi, Daniela D’Alessandro, Martha Caterina Faraguna, Andrea Boggio, Giulia Caldara, Silvia Barzaghi, Roberta Pretese, Viola Crescitelli, Serena Gasperini

**Affiliations:** 1 School of Medicine and Surgery, Università degli Studi Milano Bicocca, Milan, Italy; 2 Department of Pediatrics, Fondazione IRCCS San Gerardo Hospital, University Milano Bicocca, Monza, Italy; 3 Post-graduate program in Orthodontics, Vita-salute San Raffaele University, Milano, Italy

**Keywords:** Hypernasality, Velopharyngeal incompetence, Orthodontics, Classic infantile Pompe disease, Pompe disease

## Abstract

**Background:**

Pompe Disease is a rare lysosomal storage disorder. Although enzyme replacement therapy (ERT) has significantly extended the lifespan and improved motor function in these patients, residual orofacial muscle weakness remains as a considerable burden by affecting speech and swallowing.

**Case presentation:**

A 7-year-old girl with classic infantile Pompe disease presented with speech and swallowing difficulties. Hypernasality made intelligibility difficult. Orthodontic evaluation revealed mild anterior open bite, atypical swallowing patterns, and maxillary transverse deficiency. Radiographic assessments confirmed a Skeletal Class I relationship and the absence of all third molars. Rapid maxillary expansion was performed to correct this condition. Post-treatment, the patient showed significant improvements in speech and swallowing.

**Conclusion:**

While developing a standardized technique may not be feasible at present due to the variations in orofacial characteristics among children with Pompe Disease, incorporating orthodontic care early in its management can significantly improve functional and quality-of-life outcomes for these patients.

## Background

Glycogen storage disease type II (GSD-II), also known as Pompe Disease (PD), is a rare progressive metabolic disorder caused by autosomal recessive mutations in the gene that encodes acid α-glucosidase (GAA) located on chromosome 17q23. The deficiency of this enzyme leads to the accumulation of glycogen within cells, primarily in skeletal and cardiac muscle, as well as in other tissues like the liver and the central and peripheral nervous systems.

The phenotype of Pompe Disease consists of a wide spectrum, ranging from the most severe classic infantile form to the late onset forms in adulthood (LOPD); the entity of glycogen accumulation depends on the residual enzyme activity [1].

The most prominent features of classic infantile PD within the first few months of life are severe muscle weakness, hypertrophic cardiomyopathy, respiratory problems and failure to thrive. This severe form of the disease typically leads to death before the age of one year without treatment. In contrast, LOPD patients generally have milder and slower progression of limb weakness and respiratory difficulties, without cardiac involvement. The introduction of Enzyme Replacement Therapy (ERT) with recombinant human α-glucosidase (rhGAA), approved in Europe and in the United States in 2006, has significantly improved the overall prognosis and changed dramatically the natural history of the disease, especially for the classic infantile form [2, 3].

Both forms of Pompe Disease present an important involvement of the oral-facial muscles, leading to severe impairments in speech and swallowing [4–12], as well as impaired hearing [4, 6, 13].

The most commonly reported speech deficits in Pompe Disease include abnormal articulation, hypernasality and intelligibility. In children with Pompe Disease, speech disorders are primarily caused by velopharyngeal incompetence, which can result from muscle impairment (including the levator palatini, palatoglossus and palatopharyngeus muscles) or nerve involvement (including the cranial nerves V, VII, IX) [5, 14].

Each movement of the velopharyngeal section is determined by the coordinated activity of several muscles. For example, the levator veli palatini muscle acts as a sling to lift and retract the velum towards the posterior pharyngeal wall, while the palatoglossus muscle lowers the velum during nasal consonant production and the palatopharyngeus muscle narrows the pharynx by elevating and medializing the lateral pharyngeal walls [9].

Nervous system involvement probably also contributes to the speech disorder, resulting in a condition known as flaccid dysarthria [5, 8].

Although ERT has resulted in sustained improvement in cardiac, respiratory, and gross motor functions in patients with classic infantile Pompe Disease, varying degrees of muscle weakness still persist. Weakness in the orofacial muscles persists even in patients treated since birth with high doses of ERT, leading to ongoing and sometimes worsening speech impairments (nasal voice or rhinolalia), which represent a significant burden of the disease in terms of social relations and peer interactions [4–6].

The purpose of this article is to highlight the role of orthodontists in managing the orofacial manifestations in a child affected by classic infantile Pompe Disease.

## Case presentation

A 10 months-old female patient was diagnosed with classic infantile Pompe Disease during a viral infection.

She was born at term from non-consanguineous healthy parents by spontaneous delivery with a birth weight of 3450 g. The perinatal period was physiological and weight-stature growth always adequate for age.

At the age of 10 months hypertrophic cardiomyopathy (Left Ventricular Mass Index 237.8 g/m^2^, + 13.48 standard deviations for body surface area) was identified by running some tests during a viral infection. She also presented hepatomegaly, axial hypotonia and poor facial expression.

GAA analysis identified two severe mutations (c.525del/c.670 C > T) and Cross Reactive Immune Material (CRIM) positive status was confirmed by Western blot. The patient promptly started enzyme replacement therapy at 11 months with alpha-glucosidase 20 mg/kg/week; the dose was increased to 40 mg/kg/week at the age of 27 months because of unsatisfactory clinical findings, especially bilateral clubfoot.

The patient progressively developed hypernasality and swallowing problems as a consequence of orofacial weakness.

At the age of 7 years old, the patient was referred to an orthodontist to evaluate the oral cavity. The patient was in early mixed dentition, in line with the average for that age.

**Fig. 1:**
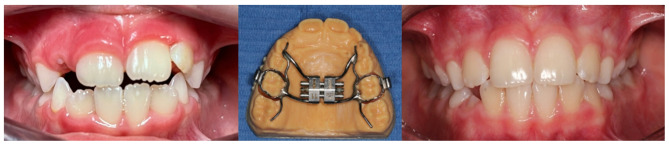
Left: before treatment (7 years old); Center: The Rapid Palatal Expander (RPE); Right: After treatment

The clinical examination showed mild anterior open bite associated with atypical swallowing, which is a myofunctional problem consisting of an altered tongue position during the act of swallowing (Fig. 1, left).

Intraoral examination showed Class I molar relationship on both sides with reduced overjet and maxillary transverse deficiency without lateral crossbite. Transverse deficiency of the maxilla is the most common skeletal problem that involves the maxilla in growing patients.

To complete the orthodontic diagnosis orthopantomography and cephalometric radiograph were taken.

Cephalometrically the patient presented Skeletal Class I relationship (SNA: 84.93°, SNB: 82.96°, ANB: 1.97°) with no vertical bone base discrepancies (SN/GoGn:32.31°, FM/GoGn:24.74°).

To improve dental occlusion and correct the transverse diameter of the maxilla, the patient underwent a rapid maxillary expansion (RME) through a rapid palatal expander (RPE), one of the most common fixed orthodontic appliances used to obtain a skeletal expansion of the maxilla (Fig. 1, center).

After an intraoral scan (Trios 3, 3Shape), a dentally anchored RPE (Hyrax type) was produced. The appliance was fixed with two occlusal rest on upper deciduous canines and with two prefabricated bands on both second upper deciduous molars.

The activation was performed once daily for 26 consequent days with a total expansion of 5 mm. The appliance then remained passively in situ for 7 months to stabilize the treatment results before the removal. The patient tolerated well the treatment. At the end of the treatment the patient had a correct transverse maxillary diameter with right overjet and overbite (Fig. 1, right).

Upper and lower midlines were coincident at the end of the treatment and I molar class was maintained on both sides.

After orthodontic intervention, the open bite was resolved. An increase in intelligibility was evident by listening to the patient speak, in particular for the occlusives and fricatives phonemes, and such findings were confirmed by the teachers and peers. Both the patient and her parents reported an important improvement in terms of quality of life. Speech therapy was not performed during the year before and after the orthodontic procedure.

## Discussion and conclusions

The lifespan and motor outcome of classic infantile Pompe patients have significantly improved, but various extents of muscle weakness remain as disease burden [2–4, 6]. Even in patients with a good response to the enzyme replacement therapy (ERT), weakness of orofacial muscles persists, and it has a great impact on daily life in terms of school and social activities [5, 8, 9]. Probably all healthcare providers working with children with classic infantile Pompe Disease have experienced difficulties in understanding what they are saying.

Facial muscle weakness is often already present in the first year of life as poor facial expression and a “tent” shaped mouth, then progresses to velopharynx involvement [5], which leads to abnormal speech and swallowing difficulties.

### Pathogenesis

Oro-facial involvement in children with Pompe Disease has been explained by:

1) Velopharyngeal dysfunction, caused by muscle impairment and cranial nerve involvement. Velopharyngeal dysfunction leads to disrupted nasal resonance (hypernasality), which can have a severe impact on the overall quality of speech, as it tends to amplify the impairments present in other parts of the speech mechanism. As previously suggested, certain articulation disorders seem to derive from velopharyngeal dysfunction rather than deficiencies in the orofacial mechanism itself [11]. For example, difficulties in producing pressure consonants (such as /p/ and /t/) and nasalizations (such as substituting /m/ for /p/ or /n/ for /d/) have been attributed to velopharyngeal dysfunction.

2) Lip incontinence: impaired strength in the muscles of the lips results in inaccurate production of bilabial consonants [15].

3) Macroglossia and a weak tongue secondary to glycogen accumulation: weakened tongue muscles impact the articulation of consonants, excluding bilabial and labiodental consonants [8]. Furtherly, a weak tongue leads to a reduced internal pressure and results in a reduced transverse dimensions of upper dental arch, which is a common feature in children with Pompe Disease [9].

4) Low tongue position: it affects the proper growth of craniofacial structures and favors dysphagia.

5) Flaccid dysarthria and involvement of the central nervous system [5, 8]: glycogen accumulates also in the central nervous system [16–19] and probably contributes to oro-facial weakness; furtherly, ERT does not cross the blood-brain-barrier.

The cause of persistence of velopharynx dysfunction in ERT-treated patient has been hypothesized as an insufficient/incomplete penetration of ERT in bulbar muscles [20].

The patient described presented velopharyngeal dysfunction, a reduced diameter of the maxilla and a low tongue position which led to difficulties in both speech and swallowing.

### Treatment

Traditional speech therapies, such as verbal exercises, have limited efficacy in addressing hypernasality [5, 8].

A recent pilot study has indicated that Continuous Positive Airway Pressure (CPAP) training could be a secure and effective alternative for children with Pompe Disease, particularly in terms of reducing hypernasality and improving speech clarity [14]. The benefits of CPAP described in this study may be explained by the resistance training on the velopharynx induced by the use of the device. So said, it did not show any benefits on articulation.

A palatal lift prosthesis or surgical interventions such as pharyngoplasty or a pharyngeal flap may be another option for these patients but have been associated to an increase in swallowing difficulties and obstructive sleep apnea [21].

### The role of orthodontists

Galeotti and colleagues conducted a detailed study on the orofacial features and the role of pediatric dentistry in the long-term management of children with Pompe Disease. The study described two cases of children aged 4 and 5 who were examined and followed for five years. The children exhibited characteristic features such as hypotonia of the facial and tongue muscles, lip incompetence, narrow palate, macroglossia, low tongue position, concave facial profile, Class III malocclusion, and mandibular prognathism. The children underwent exercises to enhance the tone and strength of the facial and tongue muscles as a part of a daily routine. Orthodontic treatment consisted of intraoral and extraoral devices. The intraoral ones were used to correct narrow palates and align the dental arches and helped expand the transverse dimension of the upper dental arch, which is often reduced in children with Pompe Disease. The extraoral devices aimed to manage the concave profile and Class III malocclusion. They provided the necessary external forces to guide the proper growth of the maxillary-malar area and mitigate mandibular prognathism. Each child’s treatment plan was tailored to their specific needs, considering the severity of their orofacial features and their response to therapy [9].

In children with classic infantile Pompe Disease, orthodontic causes of oro-facial weakness are often overlooked, such as a reduced transverse diameter of the maxilla, which is also the most common skeletal problem that involves the maxilla in growing patients [22].

Both patients reported by Galeotti as well as the patient here described presented transverse deficiency of the maxilla. The most common correction technique consists of rapid maxillary expansion (RME). The main goal of RME is to correct the transversal maxillary constriction that often results in lateral posterior crossbite and to widen the maxillary archwire.

A Rapid Palatal Expander (RPE) is an expansion screw welded to bands on upper deciduous second molars. This expansion screw must be periodically activated per day so that the resulting force causes the midpalatal suture to open and the maxillary bones diverge from each other: since the RPE provides a skeletal palatal expansion, an increase width at nasal floor also can occur [23, 24]. In fact, the activation force is capable of acting not only on the midpalatal suture, but also on the circum-maxillary sutures [25].

Numerous authors have reported several benefits of RPE to the upper airway due to the intimate anatomical relationship [21], such as increased pharyngeal dimensions, new tongue posture, changing of anatomical structures and significant improvements of nasopharyngeal functions.

Dysphagia with aspiration is also a prevalent issue in classic infantile Pompe Disease, with significant clinical implications [5, 8]. The underlying cause of dysphagia is possibly related to the same mechanism as the speech-related dysfunction, involving the progressive glycogen accumulation in the bulbar muscles and/or the lower motor neurons responsible for their control. The prevalent swallowing abnormalities observed in these patients include a delay in initiating the pharyngeal swallow, residue in the pharynx, airway invasion, weak sucking, and slow transit through the oral stage of swallowing [4, 5, 12]. In the patient described, swallowing difficulties were caused by the tongue being positioned too low, which affected the proper growth of craniofacial structures and resulted in a misaligned bite. Since dysphagia might be caused by the inability to ensure pressure of the tongue against the palate, several studies reported that RPE increases the space needed to generate the tongue pressure necessary for food bolus propulsion, thus after the maxillary expansion patients have better tongue adaptation to the widened palate and low tongue posture improves [26].

With the ongoing impact of Enzyme Replacement Therapy (ERT) and the advancements in patient management strategies and drug development, the number of children with Pompe Disease who achieve long-term survival will continue to increase with many unmet needs due to a multisystemic disorders. Despite remarkable improvements with ERT in the health and functioning of children with Pompe Disease, speech and swallowing deficits remain prevalent.

We reviewed the underlying mechanisms of speech and swallowing difficulties in Pompe disease and reported the orthodontic treatment in a case of classic infantile Pompe disease. No functional assessments were performed to document the improvement, but the positive results obtained with the orthodontic procedure were noted by the parents, peers, teachers and treating physicians.

The approach outlined in this paper serves as an illustrative example of orthodontic treatment for addressing speech and swallowing difficulties in patients with Pompe Disease. It does not aim at developing a standardized technique,, but at underlining the importance of pediatric dentistry in the management of these patients from the time of diagnosis onwards.

## Data Availability

all clinical data is present in the manuscript. Further enquiries can be addressed to the corresponding author, dr. S. Gasperini, upon reasonable request.
